# Construction of a novel risk model based on the random forest algorithm to distinguish pancreatic cancers with different prognoses and immune microenvironment features

**DOI:** 10.1080/21655979.2021.1951527

**Published:** 2021-07-09

**Authors:** Yalan Lei, Rong Tang, Jin Xu, Bo Zhang, Jiang Liu, Chen Liang, Qingcai Meng, Jie Hua, Xianjun Yu, Wei Wang, Si Shi

**Affiliations:** a Department of Pancreatic Surgery, Fudan University Shanghai Cancer Center, Shanghai, China; b Department of Oncology, Shanghai Medical College, Fudan University, Shanghai, China; c Shanghai Pancreatic Cancer Institute, Shanghai, China; d Pancreatic Cancer Institute, Fudan University, Shanghai, China

**Keywords:** Immune-related lncRNA, pancreatic cancer, random survival forest, immune infiltration, risk model

## Abstract

Immune-related long noncoding RNAs (irlncRNAs) are actively involved in regulating the immune status. This study aimed to establish a risk model of irlncRNAs and further investigate the roles of irlncRNAs in predicting prognosis and the immune landscape in pancreatic cancer. The transcriptome profiles and clinical information of 176 pancreatic cancer patients were retrieved from The Cancer Genome Atlas (TCGA). Immune-related genes (irgenes) downloaded from ImmPort were used to screen 1903 immune-related lncRNAs (irlncRNAs) using Pearson’s correlation analysis (R > 0.5; p < 0.001). Random survival forest (RSF) and survival tree analysis showed that 9 irlncRNAs were highly correlated with overall survival (OS) according to the variable importance (VIMP) and minimal depth. Next, Cox regression analysis was used to establish a risk model with 3 irlncRNAs (LINC00462, LINC01887, RP11-706C16.8) that was evaluated by Kaplan-Meier analysis, the areas under the curve (AUCs) of the receiver operating characteristics and the C-index. Additionally, we performed Cox regression analysis to establish the clinical prognostic model, which showed that the risk score was an independent prognostic factor (p < 0.001). A nomogram and calibration plots were drawn to visualize the clinical features. The Wilcoxon signed-rank test and Pearson’s correlation analysis further explored the irlncRNA signatures and immune cell infiltration, as well as the immunotherapy response.

## Graphical abstract

**Figure:**
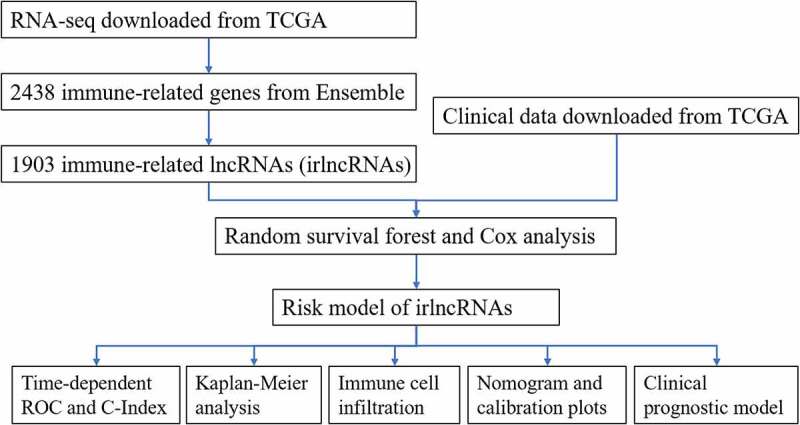

## Introduction

Pancreatic cancer is a highly malignant tumor with a 5-year survival rate of less than 10%; it is also the seventh leading cause of death in developed countries [1]. The short overall survival (OS) highlights the need for an accurate staging system to predict the prognosis, and the modification of the 8^th^ edition of the American Joint Committee on Cancer (AJCC) staging system contributes to improving prognosis prediction [2]. Surgical excision is the only option to achieve a complete cure, and chemotherapy and neoadjuvant treatment play important roles in pancreatic cancer therapy. Although immunotherapy has shown substantial improvement in several tumors, mono – or combined immune checkpoint inhibitors show limited effects in pancreatic cancer, partially due to the reduced infiltration of immune cells, poor immunogenic immune microenvironment, and abundant mesenchymal fibroblasts blocking drug delivery [3,4]. An understanding of the immune microenvironment of pancreatic cancer is required to promote its clinical application.

Long noncoding RNAs (lncRNAs) account for more than 80% of RNAs, and their transcripts are more than 200 nucleotides in length; lncRNAs do not translate into proteins. LncRNAs interfere with proteins, RNA and DNA to participate in many biological regulation processes, including transcriptome modulation and gene modification [5]. Furthermore, recent studies have delineated the mechanisms of lncRNAs that are actively involved in tumor biology, such as H19, PVT1, NEAT1 and HISLA, which were disclosed to be associated with tumorigenesis, epithelial mesenchymal transition, metastasis, chemoresistance, immune evasion and metabolic reprogramming [6–8].

Immune-related lncRNAs (irlncRNAs) have recently been studied in several cancers. Wang et al [9] identified 4 irlncRNAs to establish a risk model for lung adenocarcinoma, while 8 irlncRNAs were used to construct a prognostic model for melanoma [10]. Additionally, irlncRNAs have been utilized in glioblastoma, head and neck squamous cell carcinoma and bladder cancer [11–13]. These models demonstrate the potential clinical significance of irlncRNAs and provide novel insights to establish a clinical prognostic model.

In this article, we tried to construct a risk model using immune-related lncRNAs and demonstrated the significance of predicting prognosis using AUC and Kaplan-Meier analysis. Next, we constructed a clinical prognostic model using Cox analysis and presented it with a nomogram as well as calibration plots. Finally, we explored immune cell infiltration and responses to chemotherapy to delineate the immune landscape in pancreatic cancer patients.

## Methods

### Retrieval of the transcriptome data and identification of immune-related lncRNAs (irlncRNAs)

We obtained the transcriptome profile data (high-throughput sequencing (HTseq) counts) and clinical information of patients (n_patient_ = 176, n_normal_ = 4) from The Cancer Genome Atlas pancreatic cancer dataset (TCGA-PAAD). Next, we downloaded the gene transfer files (GTFs) from Ensemble (http://asia.ensembl.org) to annotate the transcriptome profiles and extract the lncRNA expression profiles. Additionally, immune-related genes (irgenes) were downloaded from the ImmPort database (http://www.immport.org), and 2438 genes were obtained. After Pearson’s correlation analysis of lncRNAs and irgenes, 1903 irlncRNAs were identified (r > 0.5; p < 0.001). In this step, the Hmisc package was employed.

### Establishment of the risk model by random survival forest (RSF) analysis

The RSF model was applied to determine the irlncRNAs significant to the OS and survival status according to variable importance (VIMP) and the minimal depth [14,15]. The samples were randomly divided into a training set (n = 123) and a test set (n = 53) at a ratio of 7:3. Survival tree analysis was constructed using the variables selected from the previous procedure (CTC-529P8.1, RP11-706C16.8, LINC01493, LINC01887, LINC00462, LINC01510, LINC02205, RP11-1082L8.2, RP11-402N8.1), using 1000 trees and the log-rank splitting rule. After deleting the variables with extremely low expression, the risk model was established by multivariable Cox regression analysis. Three variables (LINC00462, LINC01887, and RP11-706C16.8) were selected by Cox regression analysis and were used to predict the risk score for each sample in the training set. The forest map of the 3 irlncRNAs was drawn. Additionally, the areas under the receiver operating characteristic (ROC) curve (AUCs) for 36 months, 30 months, 24 months, 18 months, 12 months and 6 months as well as the concordance index (C-index) for the risk model were calculated. The randomForestSRC, glmnet, survival, surviminer, ggplot2, forestplot, survcopm, and prodlim packages were used in this procedure.

### Clinical validation of the risk model

According to X-Tile software (https://medicine.yale.edu/lab/rimm/research/software/), the best cutoff value for the risk score was 1.44 in the training set and 1.40 in the test set. After dividing the samples into high-risk and low-risk groups in the training set and test set separately, Kaplan-Meier analysis was conducted to analyze the difference in OS in the high – and low-risk groups. Furthermore, we drew survival plots and survival curves to visualize the difference between the groups. The survivalROC, plotROC, ggplot2, survival and survminer packages were applied in this step.

### Establishment of the clinical prognostic model

To evaluate the clinical significance of the irlncRNAs in the signature, we further conducted several analyses, including Cox regression analysis and Pearson’s correlation analysis. The AUC for each clinical characteristic and risk score were calculated to determine the potential prognostic factors. Univariate Cox regression and multivariate Cox regression analyses were applied to construct the clinical prognostic model. A nomogram was constructed, and a calibration plot was drawn to show the results of Cox analysis. The C-index of the clinical prognostic model increased from 0.599 to 0.682 after considering the risk score. The dplyr, ggolot2, ggpubr, survival, survminer, rms, and survcomp packages were used in this step.

### Exploring the correlation between the irlncRNA signature and immune cell infiltration

Correlation analysis of lncRNAs and irgenes was conducted initially. Therefore, we further explored the correlation between the irlncRNA signature and immune cell infiltration using the Tumor IMmune Estimation Resource (TIMER), CIBERSORT, XCELL, QUANTISEQ, MCPcounter and EPIC databases [16,17]. The Wilcoxon signed-rank test showed significantly infiltrated immune cells (p < 0.1), and Pearson’s correlation analysis revealed the correlation index between immune cells and the risk score. Additionally, we performed the Wilcoxon signed-rank test to assess the association between immune checkpoint inhibitor (ICI) biomarkers and risk. The Hmisc, ggplot2 and ggrepel packages were used here.

## Results

In this study, we established a risk model and clinical prognostic model using 3 irlncRNAs and explored the correlation between the irlncRNA signature and immune cell infiltration. First, correlation analysis of lncRNAs and irgenes was performed to obtain irlncRNAs. Second, random survival forest, survival tree decision and Cox regression analyses were applied to establish the risk model by irlncRNAs. Additionally, we calculated the AUC of the time-dependent ROC curve to validate its practical significance. Third, to confirm its clinical significance, Kaplan-Meier analysis, the Wilcoxon signed-rank test, and Cox regression analysis were used and confirmed that the irlncRNA signature was an independent prognostic factor among the clinical characteristics. Finally, we explored the relationship between the irlncRNA signature and immune cell infiltration, revealing that specific immune cells differentially infiltrated tissues from the high – and low-risk groups, shedding light on the immune microenvironment of pancreatic cancer. The risk model established from irlncRNAs in this study is of high predictive value. After combining the risk score into the clinical prognostic model, the C-index increased from 0.599 to 0.682, indicating that it significantly contributes to clinical prognosis efficacy among pancreatic cancer patients. Furthermore, the irlncRNA signature can predict the immune landscape, including immune cell infiltration in tumor tissue, providing insights for immunotherapy.

### Identification of immune-related long noncoding RNAs (irlncRNAs)

The process flow of this study is shown in the figure abstract. First, we obtained the transcriptome profile from high-throughput sequencing (HTseq) count data (n_patient_ = 176, n_normal_ = 4) and the clinical information of patients from The Cancer Genome Atlas pancreatic cancer dataset (TCGA-PAAD). Second, we retrieved the gene transfer files (GTFs) from Ensembl to annotate the lncRNAs from the expression matrix. Third, immune-related genes (irgenes) were downloaded from the ImmPort database. Pearson’s correlation analysis between the lncRNAs and irgenes was performed, and 1903 irlncRNAs were identified (R > 0.5; p < 0.001) (Table S1).

### Establishment of a risk model by random survival forest (RSF) analysis

**Figure 1.**
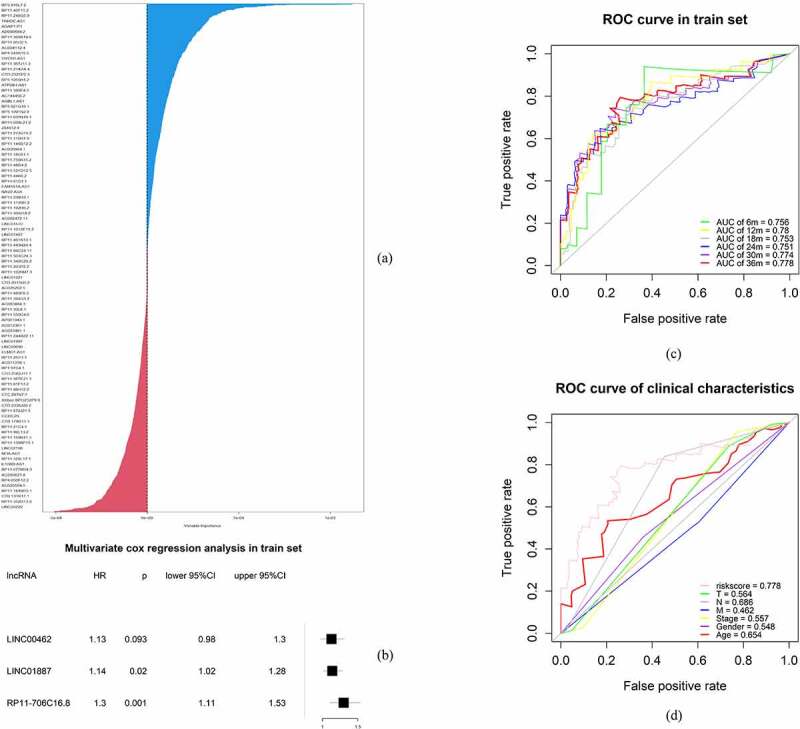
Establishment of the risk model. (a) Important variables selected using the random survival forest model. (b) Forest map of the multivariate Cox regression results. (c) ROC curve of the risk model for survival at 36 months, 30 months, 24 months, 18 months, 12 months and 6 months. (d) ROC curve of the clinical characteristics

RSF was applied to determine the irlncRNAs of most significance to the OS of pancreatic patients. In the survival tree analysis, we set trees as 1000 and the terminal node size as 3. The method was set as variable hunting with VIMP and k-fold as 5. Next, we selected the top 9 variables (CTC-529P8.1, RP11-706C16.8, LINC01493, LINC01887, LINC00462, LINC01510, LINC02205, RP11-1082 L8.2 and RP11-402N8.1) that were selected according to the variable importance (VIMP) and minimal depth. Subsequent Cox regression analysis identified 3 irlncRNAs (LINC00462, LINC01887, RP11-706C16.8), with a coefficient index and risk score for each sample in the training set calculated. To validate this model, receiver operating characteristic (ROC) curves were drawn, and the areas under the curves (AUCs) for 36 months, 30 months, 24 months, 18 months, 12 months and 6 months were 0.778, 0.774, 0.751, 0.753, 0.780 and 0.756, respectively (Figure 1). Additionally, the C-index for this risk model was 0.696 (p < 0.001).

### Clinical evaluation of the risk model

**Figure 2.**
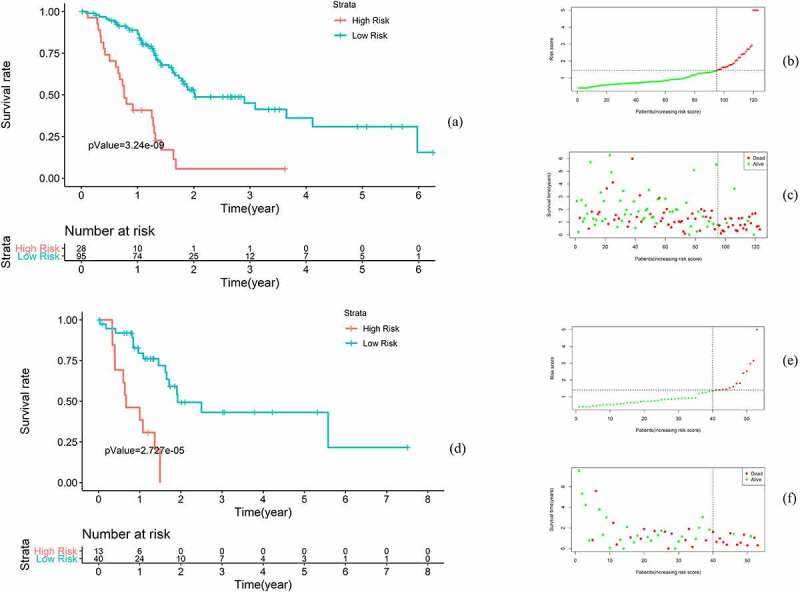
Clinical evaluation of the risk model in the training and test sets. (a-c) Kaplan-Meier analysis in the training set. (d-f) Kaplan-Meier analysis in the test set

Instead of the median risk score value, the best cutoff value (1.44) calculated using X-Tile software (https://medicine.yale.edu/lab/rimm/research/software/) was used to divide the samples into high-risk and low-risk groups in the training and validation sets. To clinically evaluate the risk model, several analytical methods were applied. First, Kaplan-Meier analysis showed that the OS of patients in the high-risk group was significantly lower than that of patients in the low-risk group (p < 0.001). In the high-risk group, the 5-year OS rate was 5.66%, the 95% confidence interval (CI) was 0.88 to 361, the 5-year OS rate was 30.9%, and the 95% CI was 18.28 to 52.4 in the low-risk group. In the test set, Kaplan-Meier analysis showed a significant difference in OS between the groups (p < 0.001) (Figure 2).

### The risk model is an independent prognostic factor for pancreatic cancer

**Figure 3.**
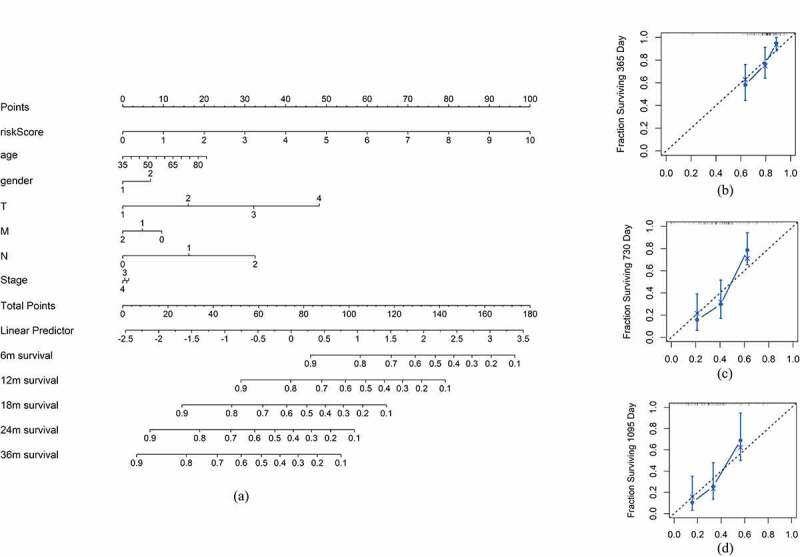
Nomogram and calibration plot of the clinical prognostic model. (a) Nomogram of the clinical prognostic model. (b-d) Calibration curves for the 1-, 3 – and 5-year survival plots comparing the actual and predicted values

To construct a more accurate clinical prognostic risk model, the AUC of each ROC for each clinical characteristic and risk score were calculated; the AUCs of the risk score, age, sex, T stage, N stage, M stage and stage were 0.778, 0.654, 0.548, 0.564, 0.686, 0.462 and 0.557, respectively, with the risk score AUC being the only one above 0.7. After univariate Cox regression analysis, the risk score (p < 0.001), N (p = 0.006) and T (p = 0.034) were selected for multivariate Cox regression analysis, revealing that the risk score (p < 0.001) and N (p < 0.05) were independent prognostic factors for pancreatic cancer (Table 1). Furthermore, the addition of the risk score to the clinical model can raise the C-index from 0.599 to 0.682, indicating that it substantially contributes to prognosis prediction. A nomogram and the related calibration plots were established to visualize the specific method, calculate the risk scores and show the ability of the model to predict OS at 6 months, 12 months and 36 months (Figure 3).

**Table 1.** Univariate and multivariate Cox regression analysis for the clinical prognostic model

Items	Univariate Cox regression			Multivariate Cox regression			C-index	
	HR	95% CI	p	HR	95% CI	P		
Risk score*	1.441	[1.250, 1.663]	<0.001	1.384	[1.195, 1.601]	<0.001	0. 682	
N*	1.997	[1.217, 3.275]	0.006	1.794	[1.017, 3.164]	0.043		0.599
T	1.829	[1.048, 3.191]	0.034	1.855	[0.977, 3.525]	0.059		
Stage	1.319	[0.829, 2.097]	0.242	– –	– –	– –	– –	– –
M	0.8561	[0.667, 1.099]	0.222	– –	– –	– –	–	–
Age	1.022	[0.996, 1.048]	0.102	– –	– –	– –	–	–
Sex	1.417	[0.865, 2.322]	0.166	– –	– –	– –	–	–

*p < 0.05

### Exploring the correlation between immune cell infiltration and the risk model

**Figure 4.**
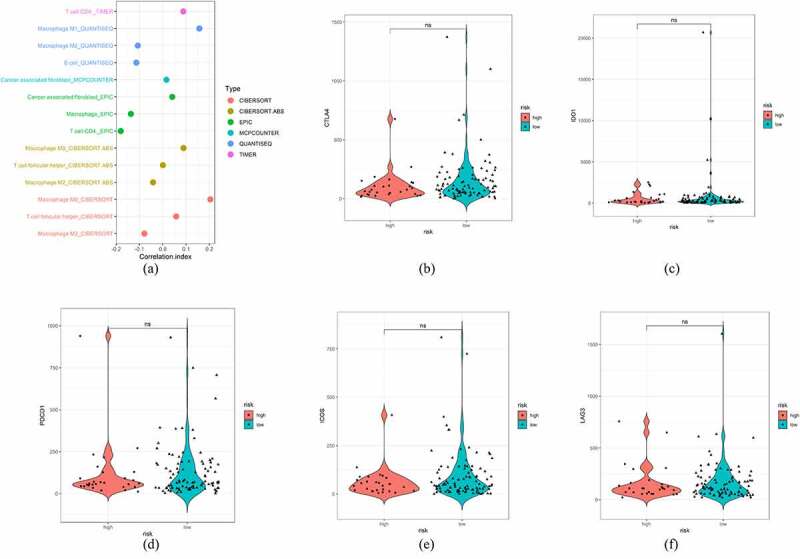
Exploration of the risk score and immune infiltration status. (a) Lollipop graph of the correlation between the immune cell infiltration status and risk score. (b-f) Violin plot of risk and ICI targets, including CTLA4, IDO1, PDCD1, ICOS, and LAG3

The irlncRNAs identified by correlation analysis of irgenes and lncRNAs at the beginning of this study may influence the immune status, such as immune cell infiltration. We uploaded the transcriptome data of lncRNAs to Tumor IMmune Estimation Resource (TIMER), CIBERSORT, QUANTISEQ, MCPcounter and EPIC resources to estimate the content of immune cell infiltration in the training set (Table S2). Next, Wilcoxon ranked analysis was used to compare the distribution of various immune cells in high-risk and low-risk patients. Wilcoxon analysis revealed that the high-risk group was associated with greater infiltration of cancer-associated fibroblasts, follicular helper T cells, CD4 + T cells, M0 macrophages and M1 macrophages, while the low-risk group was correlated with greater infiltration of B cells and M2 macrophages. Pearson’s correlation analysis was then conducted between the risk score and each infiltrated immune cell, revealing the correlation between irlncRNA signatures and infiltration of M0 macrophages and CD4 + T cells (p < 0.05) (Table S3). The correlation results were expressed in a lollipop graph. However, the Wilcoxon signed-rank test comparing risk and immune checkpoint inhibitor (ICI) biomarkers, including CTLA4, LAG3, IDO1, PDCD1 and ICOS, showed no significant association, reflecting the poor effect of ICIs in clinical trials (Figure 4).

## Discussion

lncRNAs have special localization features and functional mechanisms, including assembly with proteins, RNA and DNA to participate in various biological processes. Therefore, they have been widely applied in constructing prognostic models across tumors [18–20]. These studies also indicated that lncRNAs play an important role in tumor biological functions, including chemotherapy resistance, tumorigenesis and EMT [21–23]. Wei et al. [24] disclosed the potential biological functions of irlncRNAs in pancreatic tumors, indicating that tumor purity is negatively associated with the infiltration of fibroblasts, myeloid dendritic cells and monocytes. This finding led to further research on irlncRNAs and their potential regulatory mechanisms in pancreatic cancer. Our study focused on the potential immune functions of lncRNAs and conducted integrated clinical evaluation of the risk model. To improve the efficacy and accuracy, we validated the prognostic model in the test set. Additionally, we used multiple databases to explore the tumor immune microenvironment, revealing findings that were consistent with previous findings that lncRNAs could regulate the tumor immune microenvironment by activating immune cells and promoting immune evasion or other mechanisms [25].

The irlncRNAs identified in our study included LINC00462, LINC01887, and RP11-706C16.8. LINC00462 participates in the miR-666/TGFBR1-TGFBR2/SMAD2/3 or AKT signaling pathways to promote tumor invasion and progression in pancreatic cancer and hepatic cancer [26–28]. Additionally, BBCancer (http://bbcancer.renlab.org), an expression atlas of blood-based biomarkers across cancers, demonstrated that LINC00462 is downregulated in breast cancer and liver cancer and may be a biomarker for early diagnosis. LINC01887 is significantly downregulated in colorectal, pancreatic and liver cancer, exhibiting its potential function in gastrointestinal tumors [29]. Further study on the mechanisms of the identified irlncRNAs may reveal their potential regulatory function in the tumor microenvironment.

Although immunotherapy has substantially improved tumor treatment, it shows limited effects and many side effects in pancreatic cancer. Some clinical trials on immunotherapy, such as ICIs applied alone or combined with chemotherapy (NCT02558894, NCT02331251), immune vaccines (NCT00084383), CD40 antibodies (NCT00711191), and mesenchymal target therapy (NCT02734160), are currently underway [30–34]. In the present study, we conducted Pearson’s correlation analysis of the irlncRNA signature and common biomarkers of ICIs. However, no significant correlation was found, a finding that is consistent with the limited immunotherapy effect in pancreatic cancer [35]. Pancreatic cancer is usually regarded as poorly immunogenic and is accompanied by fewer mutated antigens recognized by patient T cells than lung cancer and melanoma [36]. However, the present study showed that high risk was associated with increased infiltration of cancer-associated fibroblasts, CD4 + T cells, M0 macrophages and M1 macrophages, while low risk was correlated with M2 macrophage infiltration, indicating that macrophage differentiation may be correlated with malignancies and may be a potential target in immunotherapy.

However, the present study is limited in the following aspects. First, the limited sample size of pancreatic cancer in TCGA and limited transcriptome data of irlncRNAs may influence the validity of the risk model. However, we applied the random survival forest model and completed integrated analysis to confirm the robustness of the risk model to improve the validity. Second, the TCGA-PAAD database lacks information on immunotherapy, limiting further evaluation of the clinical prognostic model in predicting the response to immunotherapy. Third, few experimental data support our findings, and the specific function or mechanisms of these irlncRNAs should be validated with additional experimental data. Fourth, the splitting ratio of 7:3 may introduce potential sampling bias, which could be partially overcome by conducting Cox analysis.

## Conclusions

We established a novel and robust risk model using 3 irlncRNAs and a 3-year survival AUC of 0.778 in pancreatic cancer patients. The risk model was identified as an independent prognostic factor in the clinical evaluation, and we drew the nomogram and calibration plots of the clinical prognostic model. Furthermore, the risk model could delineate the immune landscape of pancreatic cancer patients, with potential clinical significance.

## Supplementary Material

Supplemental Material

## Data Availability

The datasets supporting the conclusions of this article are available in the cancer genome atlas (http://portal.gdc.cancer.gov/), immPort (http://import.org/) and TIMER (http://timer.cistrome.org/) database.

## Research highlights:

1. Immune-related lncRNAs are closely related to pancreatic cancer prognosis and are independent prognostic factors
2. The irlncRNA risk model predicted immune cell infiltration in pancreatic cancer patients.

The AUC of the ROC curve in the risk model was higher than that in a previous study, demonstrating its robustness.
